# A Comparative Transcriptome Analysis Unveils the Mechanisms of Response in Feather Degradation by *Pseudomonas aeruginosa* Gxun-7

**DOI:** 10.3390/microorganisms12040841

**Published:** 2024-04-22

**Authors:** Chaodong Song, Rui Liu, Doudou Yin, Chenjie Xie, Ying Liang, Dengfeng Yang, Mingguo Jiang, Hongyan Zhang, Naikun Shen

**Affiliations:** 1 Guangxi Key Laboratory of Polysaccharide Materials and Modification, School of Marine Sciences and Biotechnology, Guangxi Minzu University, Nanning 530000, China; 15036104955@163.com (C.S.); liiiiiiuy@163.com (R.L.); yin13419755068@163.com (D.Y.); xiechenjie2021@163.com (C.X.); ly_ng_bjyx200112@163.com (Y.L.); mzxyjiang@163.com (M.J.); 2 Guangxi Key Laboratory of Marine Natural Products and Combinatorial Biosynthesis Chemistry, Guangxi Beibu Gulf Marine Research Center, Guangxi Academy of Sciences, No. 98, Daxue Road, Nanning 530007, China; yangdengfeng@gxas.cn

**Keywords:** *Pseudomonas aeruginosa*, feather degradation, comparative transcriptome analysis, degradation mechanism

## Abstract

Microbial degradation of feathers offers potential for bioremediation, yet the microbial response mechanisms warrant additional investigation. In prior work, *Pseudomonas aeruginosa* Gxun-7, which demonstrated robust degradation of feathers at elevated concentrations, was isolated. However, the molecular mechanism of this degradation remains only partially understood. To investigate this, we used RNA sequencing (RNA-seq) to examine the genes that were expressed differentially in *P. aeruginosa* Gxun-7 when exposed to 25 g/L of feather substrate. The RNA-seq analysis identified 5571 differentially expressed genes; of these, 795 were upregulated and 603 were downregulated. Upregulated genes primarily participated in proteolysis, amino acid, and pyruvate metabolism. Genes encoding proteases, as well as those involved in sulfur metabolism, phenazine synthesis, and type VI secretion systems, were notably elevated, highlighting their crucial function in feather decomposition. Integration of Gene Ontology (GO) and Kyoto Encyclopedia of Genes and Genomes (KEGG) taxonomies, combined with a review of the literature, led us to propose that metabolic feather degradation involves environmental activation, reducing agent secretion, protease release, peptide/amino acid uptake, and metabolic processes. Sulfite has emerged as a critical activator of keratinase catalysis, while cysteine serves as a regulatory mediator. qRT–PCR assay results for 11 selected gene subset corroborated the RNA-seq findings. This study enhances our understanding of the transcriptomic responses of *P. aeruginosa* Gxun-7 to feather degradation and offers insights into potential degradation mechanisms, thereby aiding in the formulation of effective feather waste management strategies in poultry farming.

## 1. Introduction

Feathers constitute a significant byproduct in the poultry industry, accounting for 5–7% of the total body weight of chickens [1,2]. On a global scale, around 2 million tons of feathers are produced each year as a byproduct of the poultry industry. Feathers consist of over 85% beta-keratin, along with 70% amino acids, vitamins, high-value elements, and other growth factors [3,4,5]. This composition underscores the potential use of feathers as animal feed and biofertilizers [6,7]. However, the structural integrity of feather keratin is notably robust, primarily due to the abundance of cysteine (Cys) residues linked by disulfide bonds, resulting in tightly packed structures that resist chemical and physical factors [8]. Consequently, a substantial portion of these feather wastes is either discarded or incinerated, posing significant environmental concerns [9]. Furthermore, feather waste can serve as a habitat for various pathogenic microorganisms, including *Vibrio* and *Salmonella*, while also emitting pollutants such as ammonia, nitrous oxide, and hydrogen sulfide, thereby posing risks to human health and the environment [10,11]. Consequently, the recycling of keratinous waste resources has emerged as an urgent crucial issue.

Methods for keratin hydrolysis typically include physical techniques such as pressurized hydrolysis, high-temperature puffing, and microwaving puffing, as well as chemical approaches involving acid and alkali treatments, which have traditionally been employed in the conversion of feathers into animal feed [7]. Nevertheless, these processes frequently involve significant energy consumption and can lead to the degradation of specific amino acids, such as lysine, methionine, and tryptophan. Additionally, these methods release sulfur and ammonia waste gases. Microbial keratinases offer an alternative solution by hydrolyzing rigid and highly cross-linked keratin substrates, directly targeting hydrophobic amino acid residues [4]. Consequently, the biodegradation of feather wastes by keratinolytic bacteria into valuable products, including free amino acids, peptides, and ammonium ions, has emerged as an efficient, cost-effective, and environmentally friendly approach in recent decades [12].

Microbial keratinase-producing organisms are widely distributed, including bacteria, actinomycetes, and fungi. Among these, bacteria have been the primary focus of research in the domain of keratin degradation, with *Bacillus licheniformis*, *Bacillus subtilis*, and *Bacillus amyloliquefaciens* demonstrating significant keratinase-producing capabilities [13,14]. The keratin hydrolysate resulting from keratinase activity is rich in ammonia and amino acids, presenting opportunities for the development of biological fertilizers [15]. For instance, the supplementation of animal feed with keratinase has been reported to enhance nutrient digestibility, palatability, and immune response. Nevertheless, the formidable cross-linking facilitated by disulfide bonds in keratins poses a major challenge to their degradation by proteases [16]. While keratinases, a class of proteases, are adept at breaking peptide bonds and degrading keratin, they do not effectively act on hydrophobic substrates or disrupt disulfide bonds [17]. The biodegradation of keratin substrates involves a complex process, and the underlying mechanisms are not yet fully understood. Current research suggests that keratinases often require additional enzymes or reducing agents to cleave disulfide bonds before initiating proteolysis [18]. These reduction reactions can be catalyzed by disulfide bond reductases or exogenous reducing agents. Studies on the mechanisms of disulfide bond disruption have proposed four primary theories: biomembrane potential, mechanical pressure, thiolysis, and enzymatic hydrolysis [19,20,21]. However, the precise mechanism underlying the efficient degradation of keratin by these microorganisms remains to be fully elucidated, presenting a challenge to the sustainable utilization and industrial development of keratin waste.

In our previous research, a highly efficient feather-degrading strain capable of near-complete feather degradation at 35 °C for 48 h was isolated. Nevertheless, limited knowledge exists regarding the mechanisms underlying feather degradation, particularly concerning the mechanisms involved in disulfide bond disruption [22]. Transcriptomic analysis has emerged as an indispensable and convenient tool for investigating differential gene expression. In comparison to alternative transcriptome sequencing techniques, RNA sequencing (RNA-seq) not only offers the ability to detect a broader range of expression levels with a reduced number of RNA samples but also yields highly reproducible results, allowing for both technical and biological replication.

Therefore, in the context of this study, we employed RNA-seq for transcriptomic analysis and quantitative real-time polymerase chain reaction (qRT–PCR) to assess the expression levels of key genes associated with feather degradation. Building upon the foundation of transcriptomics, we conducted a comparative analysis of genes exhibiting differential expression at crucial time points and explored the metabolic pathways implicated in feather degradation. These correlations between degradation genes and feather biodegradation pathways were established based on our prior investigations. The results of this study offer valuable insights into the mechanisms underlying feather biodegradation. These insights may prove instrumental in the engineering of strains with enhanced feather degradation capabilities and in the further development of resources for degradation enzymes.

## 2. Materials and Methods

### 2.1. Strains, Chemicals, and Media

The strain *P. aeruginosa* Gxun-7 was isolated from sludge samples obtained from a coastal duck farm in China using an enrichment culture method and was securely stored in our laboratory. It is worth noting that the GenBank accession number for this strain is MW579860.1, and it has been assigned the strain preservation number GDMCC 61615.

To activate the bacteria, they were initially cultured in LB medium for a duration of 24 h at a temperature of 35 °C. Following this incubation period, the fermentation broth was harvested at specified time intervals and subsequently subjected to centrifugation for 10 min at 13,400× *g*. The resultant crude enzyme extract obtained was utilized for subsequent experimental analyses.

### 2.2. Determination of Keratinase Activity and Feather Degradation Rate and Amino Acid

The methodology employed for assessing diagonal protease activity and feather degradation rate was adapted from Shen [22]. Mix 200 μL of fermentation supernatant with 300 μL of 2% (m/v) casein (Yuanye, Shanghai, China) substrate at pH 7.5, and react at 50 °C for 10 min. After the reaction is completed, immediately add 500 μL of 4 mol/L TCA, centrifuge at 13,400× *g* for 10 min, transfer 200 μL of the supernatant to a 2 mL centrifuge tube, add 1 mL of 0.5 mol/L NaCO_3_ (aladdin, Shanghai, China), and 200 μL of Folin’s phenol (aladdin, Shanghai, China). Mix well and react in a water bath at 50 °C for 10 min. Measure the absorbance at 660 nm using a spectrophotometer. In this context, a single keratinase unit was defined as the quantity of enzyme necessary to hydrolyze casein, resulting in the production of 1 μg of tyrosine per min at a temperature of 50 °C.

The determination of the feather degradation rate was carried out using the weight loss method. The fermentation medium underwent filtration through filter papers, and the remaining feather residue underwent thorough washing with distilled water, followed by drying and subsequent weighing to calculate the extent of weight loss. The results are presented as a percentage relative to the initial dry weight of the feather [23,24].

The fermentation broth was taken out and centrifuged at 13,400× *g* for 10 min, then treated with 5% (*v*/*v*) sulfosalicylic acid (aladdin, Shanghai, China) at a ratio of 1:8 at 4 °C for 12 h, passed through a 0.22 μm filter membrane, and the amino acids were determined by an automatic amino acid analyzer.

### 2.3. Bacterial Growth and Sample Preparation

*P. aeruginosa* Gxun-7 was added to 50 mL of non-feather medium (CG) and feather medium (TG) with three biological replicates per group. The medium of the CG was LB broth, and the medium of the TG was added: 40 g/L feather, 1.4 g/L K_2_HPO_4_, 0.7 g/L KH_2_PO_4_, and 0.5 g/L NaCl. Subsequently, bacterial cultures were collected during the exponential phase at 24 h and rinsed with phosphate-buffered saline.

Following this step, both the CG and TG samples underwent centrifugation (at 7100× *g* for 10 min) at 4 °C, followed by a washing step employing diethyl pyrocarbonate-treated water. Finally, these samples were promptly collected and subjected to flash freezing using liquid nitrogen, and they were then securely stored at a temperature of −80 °C.

### 2.4. RNA Extraction and Sequencing

Total RNA was rigorously extracted from the tissue employing TRIzol^®^ Reagent in strict accordance with the manufacturer’s instructions (Invitrogen, Waltham, MA, USA). To ensure the purity of the RNA sample, any genomic DNA present was effectively eliminated using DNase I treatment (TaKara, Shanghai, China). Subsequently, the quality of the RNA was rigorously assessed using a 2100 Bioanalyzer (Agilent Technologies, Santa Clara, CA, USA), and its concentration was accurately determined using the ND-2000 spectrophotometer (NanoDrop Technologies, Waltham, MA, USA).

For the construction of an RNA-seq transcriptome library, we rigorously followed the protocols outlined in the TruSeqTM RNA sample preparation kit from Illumina (San Diego, CA, USA). The transformation of raw images into sequences, base-calling, and the computation of quality values were conducted through the utilization of the Illumina GA pipeline, a process expertly carried out by Shanghai Majorbio Biopharm Technology Co., Ltd (Shanghai, China). This comprehensive analysis yielded 150 base pair paired-end reads. It is pertinent to note that libraries for transcriptome analysis were established employing RNA samples obtained from both the CG and TG groups.

### 2.5. Analysis of RNA-Seq Data and Feather Degradation Mechanism

The initial raw data in fastq format underwent preprocessing using Trimmomatic (version 0.36) to obtain clean reads. Subsequently, these clean reads were subjected to mapping against the NCBI Rfam databases to remove any rRNA sequences, a process carried out using Bowtie2 (Version 2.4.5) for sequence alignment.

For gene expression quantification, RNA-seq by expectation–maximization was employed, and the quantitative index utilized was the Trusted Platform Module. To identify differentially expressed genes (DEGs), the data were analyzed with DESeq2, and the criteria for DEG selection were set as Padj < 0.05 and |log_2_FC| ≥ 1 [25].

Functional annotation was performed using various tools, including EggNOG, GO, and KEGG. GO pathway enrichment analysis was executed using Goatools software (https://github.com/tanghaibao/Goatools) (accessed on 8 July 2022), while KEGG pathway enrichment analysis was conducted using KOBAS (http://kobas.cbi.pku.edu.cn/home.do) (accessed on 8 July 2022) via Fisher’s exact test. To ensure statistical rigor, p values were subjected to correction for multiple tests, with genes considered differentially expressed when the *p* < 0.05 [26].

### 2.6. Analysis by qRT–PCR and Determination of Sulfur-Containing Compound Contents

The RNA extracted for transcriptome sequencing was employed as a template for reverse transcription, with all reagents being mixed while maintaining a low-temperature environment. The resulting synthesized cDNA was subsequently used for qRT–PCR. The 16S rRNA gene served as the reference gene, and all reactions were conducted in triplicate. Details of the gene and primer sequences utilized for qRT–PCR can be found in Table S1 in the Supplementary Material. Data analysis was performed using the 2^−ΔΔCt^ method [27].

The determination of sulfite content was carried out using the pararosaniline hydrochloride method. A reaction solution was prepared by mixing 1 mL of the supernatant with 2 mL of formaldehyde–pararosaniline. After the development of a stable color, the absorbance was measured at 550 nm. The sulfite content was calculated based on a prepared Na_2_SO_3_ standard curve [28].

To assess sulfate content, barium chromate spectrophotometry was employed. Briefly, the reaction mixture consisted of 100 μL of culture supernatant, 400 μL of H_2_O, and 250 μL of BaCrO_4_. After incubation at room temperature for 30 min, 50 μL of calcium–ammonia miscible liquids and 500 μL of 95% anhydrous ethanol were added to initiate the reaction. The resulting mixture was then centrifuged at 13,400× *g* for 10 min, and the absorbance of the supernatant was measured at 420 nm. The sulfate content was determined based on a prepared Na_2_SO_4_ standard curve [29].

Furthermore, the release of sulfhydryl compounds into the fermentation medium was assessed using the method described by Ellman. Upon adding 1 mL of 10 mol/L DTNB (5,5′-Dithiobis-(2-nitrobenzoic acid)) to 500 μL of supernatant, the mixture was incubated for 10 min. Subsequently, the absorbance was measured at 412 nm after the color had stabilized, and the sulfhydryl content was calculated based on a prepared Cys standard curve [30]. The reagents used above and the configuration methods can be found in Table S5.

### 2.7. Statistical Analysis

The data underwent rigorous statistical analysis utilizing analysis of variance through SPSS software, version 17.0. To distinguish means, Duncan’s multiple range test was employed. Statistical significance was deemed at a significance level of *p* < 0.05.

## 3. Results

### 3.1. Effects of P. aeruginosa Gxun-7 on Growth and Feather Degradation

**Figure 1:**
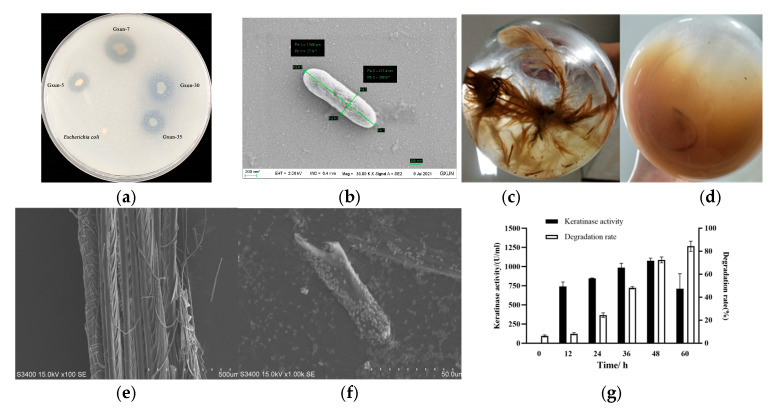
The effect of *P. aeruginosa* Gxun-7 on the degradation of feathers. (**a**): The transparent ring state of Gxun-7 on the casein-containing agar plate; (**b**): the SEM of Gxun-7, “*” indicates scale; (**c**,**d**): a comparison of feather degradation before and after Gxun-7 treatment; (**e**,**f**): the SEM of c and d; (**g**): measurement of protease activity and degradation rate in the degradation solution at different time intervals.

*P. aeruginosa* Gxun-7 displayed a distinct transparent zone on the casein plate (Figure 1a), and its morphology, as revealed by scanning electron microscopy, exhibited a spore-free, rod-shaped structure (Figure 1b). Following incubation at 35 °C for 48 h, a noticeable degradation of feathers was evident, with a feather degradation rate reaching as high as 82.6% (Figure 1c,d). Scanning electron microscopy images further depicted the significant breakdown and disassembly of feathers (Figure 1e,f).

Keratinase activity exhibited a gradual increase, reaching its maximum at the 48 h mark after Gxun-7 inoculation, with the highest recorded keratinase activity being 1075.3 U/mL. As keratinase activity escalated, so did the rate of feather degradation, indicating a direct proportional relationship between feather degradation and keratinase activity (Figure 1g). These findings underscore the remarkable feather-degrading capabilities of *P. aeruginosa* Gxun-7. However, the precise mechanisms underlying feather degradation remain poorly understood, thereby impeding the widespread application of this strain on a large scale.

### 3.2. RNA-seq Analysis

**Figure 2:**
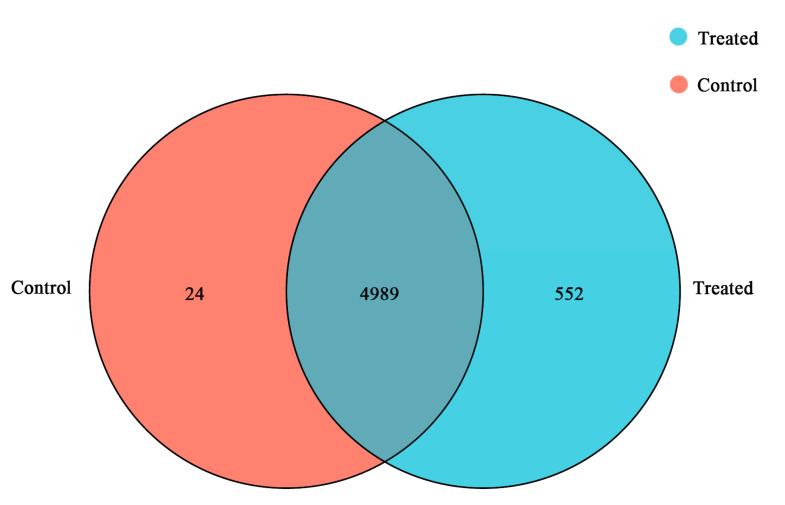
The Venn diagram illustrates gene differential expression. The total number of genes detected as expressed for each sample is represented by the sum within each circle, while the overlap between circles signifies genes expressed in both tested samples.

After filtration, over 30 million high-quality reads were obtained. The error rate for each sample was found to be less than 0.0258%. Additionally, the quality metrics Q20 and Q30 exceeded 97.36 and 93.73, respectively, indicating the reliability of the transcriptome sequencing data (refer to Table S2). A total of 5013 expressed genes were identified in the CG, while 5541 were identified in the TG. Among these genes, 4989 were co-expressed in both samples. Notably, 24 genes were found to be specifically expressed in CG, while 552 genes were specific to TG (Figure 2).

**Figure 3:**
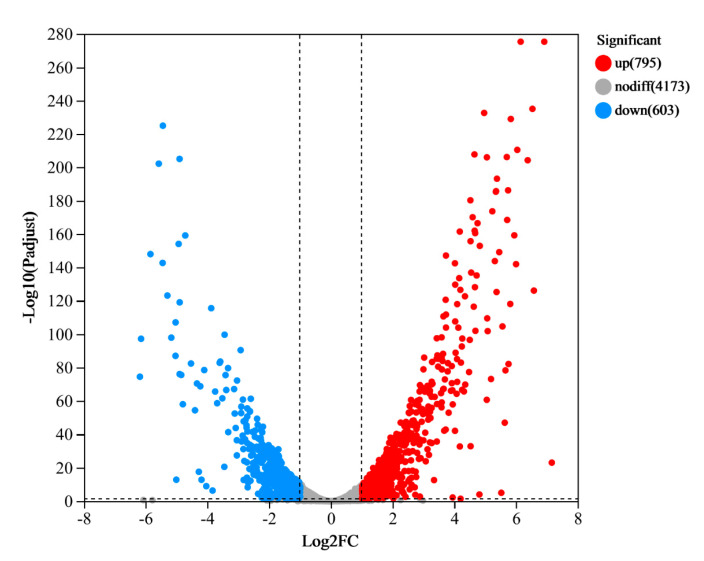
The volcano plot illustrates the differentially expressed genes (DEGs) between the treated group (TG) and the control group (CG). The y-axis represents the mean expression value of log10 (p-adjust), while the x-axis displays the log2FC value. Gray dots indicate non-differentially expressed genes, red dots represent upregulated genes, and blue dots denote downregulated genes.

To assess the degree of similarity between these samples, we calculated both correlation and variance. In this study, we utilized principal component analysis (PCA) and a heatmap to visualize this correlation. As illustrated in the heatmap and PCA plots (Figures S1 and S2), there was a high degree of correlation between the CG and TG samples, underscoring the accuracy of the sampling process. The gene cluster heat map analysis showed that the gene expression of the samples was well differentiated (Figure S3). DESeq2 was employed to assess DEGs between the samples in comparison to CG. In the TG, a total of 438 genes exhibited significant differential expression, comprising 282 genes that were significantly upregulated (|Log_2_FC| ≥ 1 and Padj ≤ 0.05) and 156 genes that were significantly downregulated (|Log_2_FC| ≤ -1 and Padj ≤ 0.05) (Figure 3).

### 3.3. Functional Enrichment Analysis of DEGs in P. aeruginosa Gxun-7

**Figure 4:**
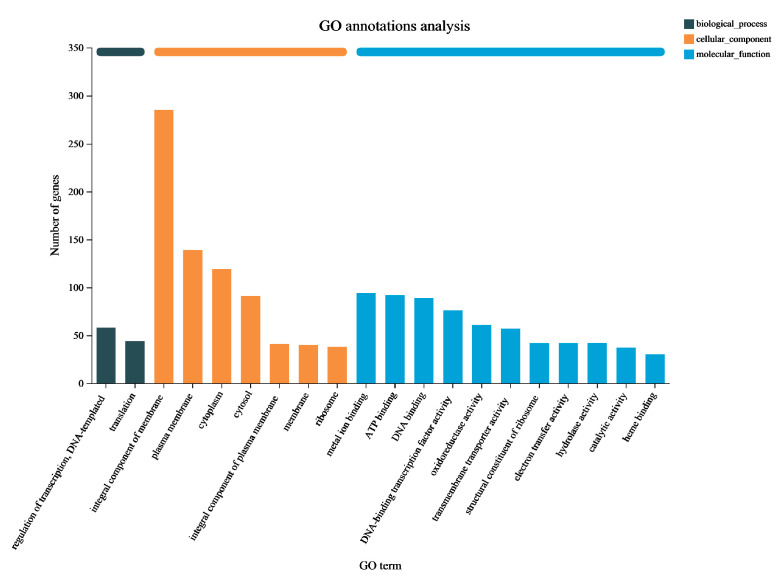
Gene Ontology (GO) function classification of DEGs. The abscissa represents the primary functional type, and the ordinate represents the number of DEGs.

The Gene Ontology (GO) classification system serves as an international framework for standardizing and describing the attributes of genes and gene products across various organisms. In this analysis, all DEGs were classified into three categories based on their respective biological processes, cellular components, and molecular functions, accounting for 102 (6.72%), 753 (49.64%), and 662 (43.64%) genes, respectively (Figure 4). In terms of “biological process”, 58 genes were found to be related to DNA template and transcriptional regulation (GO: 0006355), while 44 genes were associated with translation (GO: 0006412). The highest number of DEGs in the “cellular component” were observed in the membrane (GO: 0016021), with a total of 285 genes. Within the “molecular process” category, the top three classifications were metal ion binding (GO: 0046872), ATP binding (GO: 0005524), and DNA binding (GO: 0003677), with 94, 92, and 89 DEGs, respectively. Collectively, these data suggests that the metabolic functions of *P. aeruginosa* Gxun-7 were influenced following exposure to feathers, and the DEGs associated with these GO terms may play a role in the degradation process of feathers by the Gxun-7.

**Figure 5:**
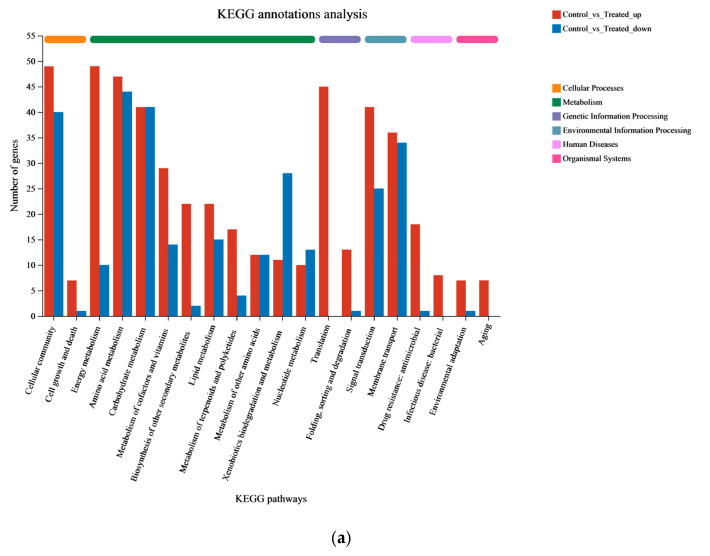

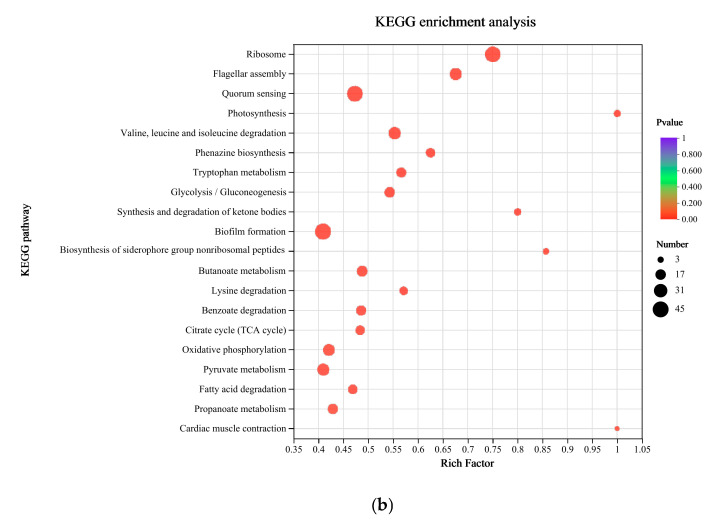
Pathway classification map of significantly enriched DEGs. (**a**): KEGG annotation results. The abscissa represents the pathway type, and the ordinate represents the number of DEGs. Red indicates upregulated genes and blue indicates downregulated genes. (**b**): KEGG enrichment results. The abscissa represents the enrichment rate, and the ordinate represents the pathway type. Bubble size indicates the number of differentially expressed genes, and bubble color indicates significance.

Pathway-based analysis plays a vital role in revealing the biological functions of genes. To further explore the potential roles of DEGs, KEGG pathway analysis was conducted to identify the metabolic pathways enriched in the process of feather degradation. The results indicate that out of the 272 distinct metabolic pathways identified between the CG and TG, they can be categorized into six major groups: metabolism, genetic information processing, environmental information processing, cellular processes, organismal systems, and human diseases. These categories comprised 123, 4, 59, 76, 5, and 5 different metabolic pathways, respectively. A total of 446 DEGs (57.18%) were associated with metabolic pathways, with environmental information processing involving 139 genes (17.82%). The amino acid metabolism pathway exhibited the greatest number of upregulated and downregulated genes, with 47 and 44 DEGs, respectively, followed by the cellular community metabolism and carbohydrate metabolism pathways with 89 and 80 DEGs (Figure 5a). A total of 16 of the top 20 differential pathways were related to metabolism, suggesting that Gxun-7 adopts an alternative metabolic strategy when utilizing feathers as its exclusive carbon and nitrogen source compared to a medium without feathers. The remaining 4 pathways in the top 20 included flagellar assembly, quorum sensing, oxidative phosphorylation, and the bacterial secretion system, indicating the functions of Gxun-7 in environmental sensing, effective oxidative respiration, and material transport during feather degradation (Figure 5b).

Furthermore, 141 DEGs were annotated into the amino acid anabolic pathway, encompassing various pathways such as “Valine, leucine and isoleucine degradation” (map00280) with 26 DEGs, “Tryptophan metabolism” (map00380) with 17 DEGs, “Glycine, serine and threonine metabolism” (map00260) with 14 DEGs, “Lysine degradation” (map00310) with 12 DEGs, “Glutathione metabolism” (map00480) with 7 DEGs, “Cysteine and methionine metabolism” (map00270) with 7 DEGs, and more. This indicates that the synthesis and metabolism of amino acids play a crucial role throughout the entire biological process. Moreover, 40 DEGs were annotated into “ABC transporters” (map02010). ABC transporters are essential for detoxification as they excrete toxic substances. Notably, the transporter genes of sulfate/thiosulfate, zinc, phosphate, phosphonate, nucleoside, and amino acid exhibited significant changes. The activity of these transporters highlights the importance of these substances. It is worth mentioning that the pathways “sulfur metabolism” (map00920) and “sulfur relay system” (map04122) appear to be potentially related to sulfite formation, warranting further investigation. Relatively fewer DEGs were annotated in organismal systems and human disease pathways, with 16 and 27 genes, respectively.

### 3.4. Transcriptome Analysis of the Gene Expression Differences in Cells of CG and TG

After performing GO and KEGG correlation analyses, we identified 20 key genes that could potentially be associated with feather degradation (Table 1). These genes were found to be associated with protease synthesis, pyocyanine biosynthesis, chitinase and catalase synthesis, amino acid metabolism, sulfite synthesis, and metabolism. The upregulated genes were mainly extracellular proteases, metabolism-related genes, transmembrane transport-related genes, and membrane proteins. Type Ⅵ secretion system protein and keratinase coding gene (*kp2*, NCBI accession number: OM992359) were found to be the two most upregulated with Log_2_FC 7.15 and 6.91, respectively. Furthermore, the genes of reductase and phenozine metabolism related were also significantly upregulated (Table 1). These suggest that the addition of feathers to the medium acts as an inducer, stimulating the expression of the *tssA*, *kp2*, *katA,* and ribosomal gene in *P. aeruginosa* Gxun-7, ultimately promoting the synthesis of keratinase and facilitating feather hydrolysis. However, their specific roles in feather degradation is still unknown and requires further investigation. The results of the analysis on differential gene expression show a significant upregulation of the keratinase expression gene, *kp*2, indicating that the bacterium synthesizes a large amount of keratinase to degrade keratin in feathers. Meanwhile, the upregulation of genes related to the type Ⅵ secretion systems is also significant (Log_2_FC = 7.15), which assists in the secretion of keratins. Therefore, the enhanced expression of this efflux pump could transport more phenazines out of the cell.

Moreover, the gene expression levels of catalase and superoxide dismutase (SOD) increased along with the increase in phenazines production, which has been demonstrated to increase the levels of ROS [31]. Extracellular proteases degrade degradable proteins into short peptides and partial amino acids outside the cell, which are then transported into the cell for further degradation into amino acids. Disulfide bonds are critical for keratin degradation, so breaking the disulfide bonds is a key step in keratin degradation [19,21].

**Figure 6:**
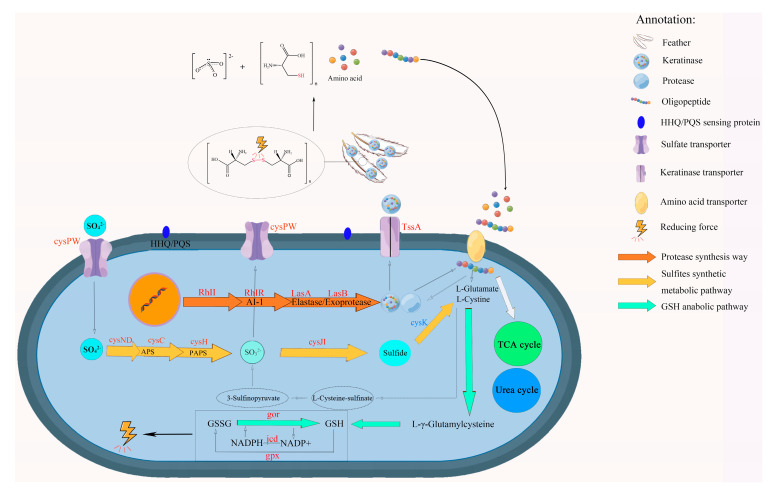
Hypothetical model of *P. aeruginosa* Gxun-7 feather utilization mechanisms. The color red indicates that the gene is upregulated and the color blue is downregulated. (GSH: reduced glutathione; GSSG: oxidized glutathione; APS: Adenosine 5′-phosphosulfate; PAPS: 3′-Phosphoadenosine 5′-phosphosulfate); the relevant genetic information is provided in Supplementary Table S3.

The experimental results presented above suggest that the degradation of feathers by *P. aeruginosa* Gxun-7 is likely a complex mechanism involving several factors. This includes the enzymatic action of various proteases, mainly keratinases, as well as the action of sulfite and the reducing power provided by the bacterium. The degradation pathways of feather by Gxun-7 obtained from transcriptome analysis and pathway enrichment are illustrated in Figure 6.

### 3.5. Results of Sulfate, Sulfite, and Sulfur-Containing Compounds

**Figure 7:**
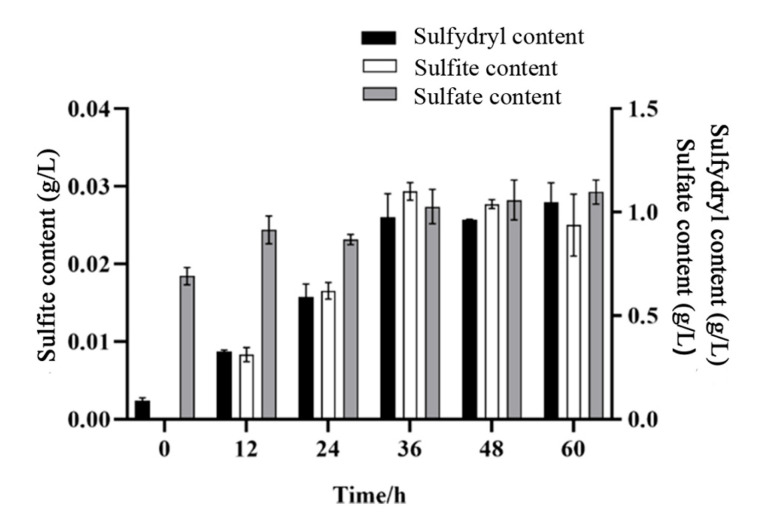
Changes in sulfates, sulfites, and sulfydryl-containing compounds during degradation at different times.

Generally, the degradation process of feather keratins causes changes in the content of sulfur-containing compounds. In the present study, we analyzed the levels of sulfur-containing compounds, specifically sulfate and sulfite, at various time intervals (12, 24, 36, 48, 60 h) during the fermentation of feathers (Figure 7). Initially, the medium contained only sulfate but no sulfite. With the proceeding of feather degradation, the content of sulfate and sulfur-containing compounds concentration increased continuously in the whole degradation period. However, the content of sulfite reached a maximum of 0.03 g/L at 36 h, and then began to decrease with the extension of fermentation time. This suggests that *P. aeruginosa* Gxun-7 undergoes a transformation among sulfate, sulfite, and sulfur-containing compounds during the degradation of feathers.

### 3.6. Validation by qRT–PCR

**Figure 8:**
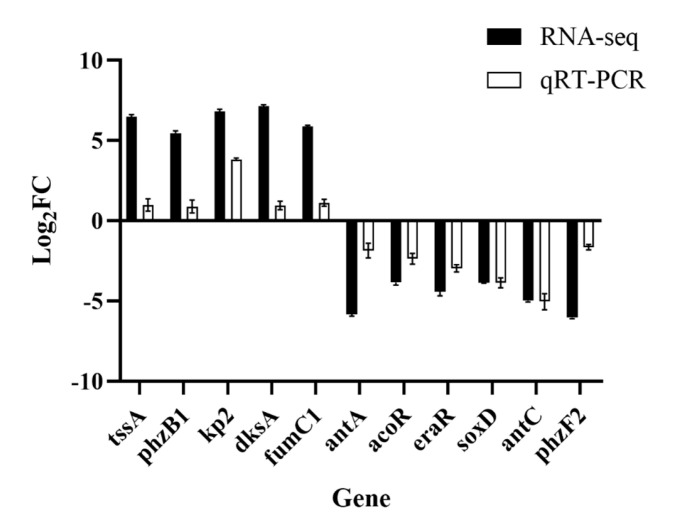
Validation of RNA-seq data by qRT–PCR. Eleven DEGs involved in the feather degradation by Gxun-7 were selected for verification. The data are expressed as the mean fold change relative to the control samples.

Eleven genes involved in the feather degradation pathway were selected for further verification using qRT–PCR analysis. One of the prominently upregulated genes is *kp2*, which plays a pivotal role in the synthesis of keratinase. This observation suggests a significant increase in the synthesis of keratinase in the TG. The *phz* genes are associated with the biosynthesis of pyocyanine, which is a specific pathway in *P. aeruginosa*. Moreover, this pathway plays a crucial role in the metabolic fate of amino acids [31]. The upregulation of chitinase genes may be linked to feather shafts, contributing to feather keratinization. Additionally, genes involved in sulfite synthesis and metabolism exhibited upregulation, indicating the possible presence of sulfite in the TG. The presence of sulfite has a profound effect on the disulfide bond formation in feathers. The thiolysis theory, recognized by most scholars in studies on feather degradation, emphasizes the significance of sulfite as an essential substance. Figure 8 shows the relative transcription level of selected genes in both RNA-seq data and qRT–PCR. The result is consistent with RNA-seq data and confirms the upregulation and downregulation of the feather degradation genes in the *P. aeruginosa* Gxun-7.

## 4. Discussion

The feature that renders keratins highly resistant to degradation by proteases is their extensive cross-linking through disulfide bonds. The biodegradation of keratin substrates is a complex process, and its mechanism remains incompletely understood. According to current research, effective keratin degradation not only involves the action of keratinases but also necessitates the disruption of disulfide bonds through specific redox mechanisms involving reductases or reductants. [32]. Our previous study demonstrates that *P. aeruginosa* Gxun-7 is a highly effective feather-degrading strain [22]. Therefore, our research employed transcriptome analysis to investigate how feather exposure influences gene expression and cellular metabolism.

The denaturation of keratin by the reduction of its disulfide bonds is the first step in feather keratin degradation. Sulfite (SO_3_^2−^) is a potent reducing agent and is capable of breaking the disulfide bonds present in keratin, making it more susceptible to degradation by keratinases [1]. Microorganisms release sulfites into the environment while degrading keratin, as seen in dermatophytes and *Streptomyces sp.* In this process, cysteine undergoes sulfonation through cysteine dioxygenases (Cdos), and the reducing agent sulfite disrupts the S–S bond in keratin, yielding L-cysteine and thiocysteine. This, in turn, leads to the denaturation of keratin, thereby facilitating its degradation [33]. Sulfite and free cysteinyl groups are secreted to reduce the disulfide bonds in the feather keratin, allowing proteases to access the peptide bonds of keratin. This process hydrolyzes the bonds, generating peptides and amino acids. These breakdown products are then imported into the cell through upregulated peptide and amino acid transporters. Within the cell, peptides may undergo further hydrolysis by intracellular proteases. Cysteine dioxygenases (CDOs), enzymes that catalyze the sulfoxidation of cysteine to cysteine sulfinic acid, have been extensively researched in eukaryotes due to their involvement in various diseases [34]. Contrastingly, only a limited number of prokaryotic enzymes of this category have undergone investigation. In *Pseudomonas aeruginosa*, two homologues of cysteine dioxygenase (Cdo and p3MDO) have been previously identified. Nevertheless, no alterations in genes associated with L-cysteine–sulfinate were discerned from our transcriptome data. Consequently, we posit the existence of alternative pathways in strain Gxun-7 for sulfite production.

The genes (cysNC, cysC, csyH, and cysJI) involved in sulfate transport and metabolism to sulfite are upregulated in the transcriptome of TG. In the previous study, we also found that adding 0.1g/L ZnSO_4_ in the medium could significantly improve the feather degradation effect. Peng also found that the external addition of sulfites could further promote feather degradation [35]. It may be that a high concentration of sulfate in the environment inhibited metabolic pathways related to Cdo of Gxun-7. Therefore, the sulfite formation mechanism of this strain may be as follows: extracellular sulfates are transferred into the cells and converted to adenylyl sulfate (APS) through the action of sulfate transfer protein (CysFUWA), followed by further conversion to 3′-Phospho-5′-adenylyl sulfate (PAPS) by APS synthase and reduction to sulfite by phosphoadenosine phosphosulfate reductase. Sulfite is then excreted to the outside, further contributing to the reduction of disulfide bonds in feathers. In order to prevent damage to cells caused by excessive sulfites, the strain reduced sulfites to sulfides by upregulating the sulfite reductase gene (*Sir*) [36]. The above-mentioned genes showed upregulated expression in the comparative transcriptome, suggesting that these pathways may play important roles in disulfide bond reduction (Figure 6). Furthermore, sulfite was also detected in the fermentation broth, indicating the presence of thiolysis in the feather degradation by *P. aeruginosa* Gxun-7 (Figure 7).

Previous study has also identified the role of γ-glutamyl transpeptidase-glutathione (GGT-GSH) serving as a redox principle during degradation of feather keratin by recombinant *Escherichia coli* harboring keratinase from *P. aeruginosa* KS-1 [37]. The γ-glutamyl transpeptidase (GGT) in the periplasm generates a free cysteinyl group, acting as a potent reductant that enhances the sulfitolysis of resistant proteins, rendering them more susceptible to degradation by keratinases. GGT catalyzes the hydrolysis of glutathione (GSH) to form cysteinyl–glycine, a significantly more robust reductant for feather breakdown compared to glutathione alone [38]. GGT is also an important component of the γ-glutamyl cycle. The genes (gor, gpx, and icd) involved in the GSH pathway, which can form a conversion cycle with oxidized glutathione (GSSG) were discovered to be significantly upregulation in the transcriptome of TG. Furthermore, by analyzing the amino acid content in the fermentation broth, the concentrations of Cys, Glu, and Gly, which are critical substances for GSH formation, were unexpectedly low in the fermentation broth (Table S4). Meanwhile, during the GSH and GSSG conversion process, glutathione reductase (GR), which possesses disulfide bond reductase activity, may also play an important role in disulfide bond cleavage. GR has also been reported to have disulfide bond reductase activity, which may also play a major role in disulfide bond cleavage [33,39]. The related mechanisms of glutathione synthesis and feather metabolism may be as follows: Glu and Cys are first converted to γ-glutamylcysteine by the action of glutamate–cysteine ligase (GCLC), next, γ-glutamylcysteine and Gly are reduced to GSH under the action of glutathione synthase (GSS). GSH has strong reducing power and can be interconverted with its oxidized state glutathione disulfide (GSSG) through the actions of glutathione peroxidase (gpx) and glutathione reductase (gor) (Figure S5). Meanwhile, GSH also is hydrolyzed by GGT to produce cysteinyl–glycine and γ-glutamylcysteine [40]. Therefore, another possible mechanism by which the strain Gxun-7 disrupt disulfide bonds of feather keratins is secreting reducing agents like cysteinyl–glycine.

The gene cluster *phzABCDEFG* encodes enzymes that catalyze the conversion of chorismate, the end product of the shikimate pathway, into PCA (phenazine-1-carboxylic acid), which serves as the precursor for synthesizing phenazine derivatives [41]. Transcriptome analysis indicated that the gene expression level of phenazine biosynthesis cluster (*phzABCDEFG*) and resistance to reactive oxygen species (ROS) (*sodM* and *katAB*) increased significantly by the addition of feather comparing to the TG group (Table 1). In addition, the phenazine content in feather degradation solution increased with the increase in fermentation time. The possible mechanism may be after a long-period reduction of disulfide bonds in feathers causes an increase in ROS levels in the cell. In order to avoid the damage of ROS, cells reduce the level of ROS by upregulating related scavenger enzyme genes such as superoxide dismutase (SOD), catalases, peroxidases, and dismutases. It has been reported that SOD, catalases, and peroxidases, which can convert ROS into less harmful products to reduce the damaging effect of hydrogen peroxide on cells, play critical roles in cell resistance to ROS [42]. SOD is a crucial component of the microbial redox system, serving as a vital scavenger of reactive oxygen species (ROS) within the cell. It plays a pivotal role in the cellular antioxidant system, safeguarding the cell from damage [43]. Catalase catalyzes the breakdown of hydrogen peroxide into water and oxygen, thereby safeguarding cells against oxidative damage [44]. Therefore, the addition of feather increased the expression of genes involved in ROS resistance and resulted in lower level of ROS. Concurrently, there was a notable decrease in the levels of tyrosine, phenylalanine, and tryptophan, synthesized from chorismate, upon the addition of feather. These findings indicate that the inclusion of feather heightened the carbon flux directed towards the biosynthesis of phenazines, thereby facilitating an increase in phenazine production.

Bacterial type VI secretion systems (T6SS) are widely distributed among Gram-negative bacteria and serve as a versatile protein export mechanism that transports effectors into eukaryotic or prokaryotic target cells. Additionally, T6SS plays a role in metal ion uptake, including iron, manganese, and zin [45,46,47,48], conferring an advantage during bacteria–bacteria competition. *P. aeruginosa* possesses three T6SSs, designated H1-, H2-, and H3-T6SS. Thirteen essential genes are conserved in all T6SSs [49]. Generally, T6SS are composed of 13–15 structural proteins divided into three interlocking structures: the intermembrane anchor, the baseplate, and the needle/sheath. The T6SS secretes two categories of proteins, valine–glycine repeat protein G (VgrG) and hemolysin coregulated protein (Hcp). The Hcp protein shares structural similarities with a phage tail tube component, while VgrG proteins exhibit resemblance to the puncturing device located at the tip of the phage tube [50]. It has been suggested that the primary role of Hcp is the formation of nanotubes on the bacterial surface, which could facilitate the transportation of other effector proteins dependent on the T6SS. Conversely, VgrG proteins assemble into trimeric complexes that potentially function as puncturing devices, enabling the perforation of membranes to facilitate the passage of proteins or macromolecular complexes [48]. Notably, Hcp is considered as the hallmark of T6SS and can be used as a biomarker for activation of T6SS. The expression of T6SS in *P. aeruginosa* is regulated by the quorum sensing (QS) system [51]. QS is crucial for collective adaptive responses and regulates both bacterial virulence and biofilm formation. *Pseudomonas aeruginosa* possesses multiple QS systems, including two N-acyl-homoserine lactone-based systems (las and rhl systems) and one quinolone PQS system (pqs) [47]. Transcriptome analysis indicated that the genes expression level of T6SS structural proteins and QS systems (*sodM* and *katA*) increased significantly with the addition of feather comparing to the TG group. Thus, the synthesized keratinase, phenazine, and reducing agents, such as sulfite and cysteinyl–glycine, can be quickly removed from the cell.

## 5. Conclusions

In summary, this study provides deep insights into the molecular mechanisms of response in feather degradation by *P. aeruginosa* Gxun-7 through comprehensive biochemical and transcriptomics analyses. The RNA-seq analysis identified 5571 differentially expressed genes; of these, 795 were upregulated and 603 were downregulated. Upregulated genes primarily participated in proteolysis, amino acid, and pyruvate metabolism. This investigation culminated in the synthesis of a hypothetical degradation mechanism: under the activation of QS signals, Gxun-7 produces a large amount of proteases, including keratin, which first degrade keratins with broken disulfide bonds into peptides and amino acids. Extracellular sulfates, peptides, and amino acids are transported into the cells through T6SS. The sulfate is converted into sulfite inside the cell, which is further transported outside the cell to break the disulfide bond of keratin. The abundant Cys, Glu, and Gly participate in cellular metabolism, and synthesis of GSH. Next, the reductive cleavage of disulfide bonds in feather by the interaction of oxidation, sulfite, GSH, and cysteinyl–glycine, which loosens the structure of keratin, enhances its solubility, and exposes more protease attack sites. Finally, the remaining loose structure is completely degraded by keratinase. This study enhances our understanding of the transcriptomic responses of *P. aeruginosa* Gxun-7 to feather degradation and offers insights into potential degradation mechanisms, thereby aiding in the formulation of effective feather waste management strategies in poultry farming.

## Figures and Tables

**Table 1:** Table of key gene screening.

Gene	Gene Description	Log_2_FC
*tssA*	Type Ⅵ secretion system protein	7.15
*kp2*	Keratinase KP2	6.91
*ahpF*	Alkyl hydroperoxide reductase subunit F	6.15
*phzB1*	Phenazine biosynthesis protein	5.76
*dhcB*	Dehydrocarnitine CoA transferase C subunit B	5.71
*phzA1*	Phenazine biosynthesis protein	5.63
*phzC2*	Phenazine biosynthesis protein	5.52
*dhcA*	Dehydrocarnitine CoA transferase C subunit A	5.34
*pchD*	Pyochelin biosynthesis protein	4.97
*sodM*	Superoxide dismutase	4.82
*phzF1*	Phenazine biosynthesis protein	4.81
*phzM*	Phenazine-specific methyltransferase	4.72
*katA*	Catalase	4.68
*pchE*	Dihydroaeruginoic acid synthetase	4.65
*phzD1*	Phenazine biosynthesis protein	4.53
*katB*	Catalase	4.21
*chiC*	Chitinase	4.12
*lasA*	Protease precursor	2.87
*sir2*	Sulfite reductase	1.61
*gor*	Glutathione reductase	1.18

## Data Availability

Raw transcriptome data have been uploaded to National Center for Biotechnology Information (NCBI) (Accession SRA number: PRJNA1005561). All the data presented in this study are available on request from the corresponding author.

## Supplementary Materials

The following supporting information can be downloaded at: https://www.mdpi.com/article/10.3390/microorganisms12040841/s1, Figure S1: An analysis of the sample correlation heat map; Figure S2: Principal component analysis (PCA) correlation analysis was conducted to examine the correlation levels between samples; Figure S3: Gene cluster heatmap analysis; Figure S4: Schematic representation of sulfite metabolism; Figure S5: Schematic representation of GSH production; Table S1: Primer sequences; Table S2: Statistical table for quality control of sequencing; Table S3: Relevant major genetic information in Figure 6; Table S4: Analysis of amino acid species and contents before and after degradation; Table S5: Preparation table of main reagents.
