# Supplementary material for: A Comparative Transcriptome Analysis Unveils the Mechanisms of Response in Feather Degradation by Pseudomonas aeruginosa Gxun-7
Source: Microorganisms. 2024 Apr 22;12(4):841. doi: 10.3390/microorganisms12040841 (PMC11052024; doi:10.3390/microorganisms12040841)
Supplement: Supplementary file 1 [file microorganisms-12-00841-s001.zip › microorganisms-2928015-supplementary.pdf]

Table S1. Primer sequences.

Gene ID	Gene Name	Forward primer (5′→3′)	Reverse primer (5′→3′)
PA1656	tssA	ATGACCTATTCGAGCAAGCT	AGGATTCCCGTTGGTAGAGG
PA4211	phzB1	ATGCCTGATACGACAAATCC	GTTGTACCACTCCCAATCGG
PA3724	kp2	ATGAAGAAGGTTTCTACGCT	GTTGTGGAATTGCTCGTAGC
PA5536	dskA	ATGACCGAACAGGAACTGCT	AGCGCTTCGTCGATCTTGTC
PA4470	fumC1	ATGACTGACACCCGCATCGA	AGTCCTCCTGCAGCAGTTGC
PA2512	antA	ATGAACGCTACCCGCAGAAG	GTTGACCAGCGCGTGCAACT
PA4147	acoR	ATGCTTTCCGCACACTCGAA	ATGCAGTCGCGCCACCTCTT
PA1980	eraR	GTCCTGCTGGTGGACGATCATTTCG	AACAGCACACGCAACTGCGG
PA5417	soxD	GGCGAACTGCGTTCCGAAGA	TGGCGGGTGACGTTGAAGTA
PA4151	antC	GCGAGAAAGATCAGCTACCA	TGGTAGAGGCCCTTGGTGAC
PA2514	phzF2	AATCACAAAGTCGCCCTCAG	GTAGTCCAGGCTGTAGCTGC

Table S2. Statistical table for quality control of sequencing.

Sample Name	Raw reads	Raw Bases (bp)	Clean Bases (bp)	Clean Error Rate (%)	Clean Q20(%)	Clean Q30(%)
Treated_3	30845134	4657615234	4200719637	0.0252	97.68	94.27
Treated_2	29790196	4498319596	4082015281	0.0249	97.86	94.51
Treated_1	29843346	4506345246	4104023137	0.0247	97.96	94.69
Control_3	30785212	4648567012	4020128007	0.0257	97.41	93.82
Control_2	27849154	4205222254	3587167084	0.0258	97.36	93.73
Control_1	29073348	4390075548	3789502480	0.0257	97.44	93.84

Table S3. Relevant major genetic information in Figure 6.

Gene	Gene description	Log_2FC	up/down
cysP	sulfate-binding protein of ABC transporter	1.43	up
cysU	sulfate-binding protein of ABC transporter	1.26	up
cysW	sulfate transport protein CysW	1.14	up
cysNC	Assimilatory sulfate reduction, sulfate => H2S	0.41	up
cysC	Assimilatory sulfate reduction, sulfate => H2S	0.64	up
cysH	phosphoadenylyl-sulfate reductase	1.12	up
cysJ	oxidoreductase	1.26	up
cysI	sulfite or nitrite reductase	1.61	up
cysK	cysteine synthase A	-1.02	down
gor	glutathione reductase	1.18	up
gpx	glutathione peroxidase	1.08	up
icd	isocitrate dehydrogenase	1.23	up
rhlI	autoinducer synthesis protein	1.36	up
lasA	LasA protease precursor	2.67	up
lasB	Elastase	6.91	up

Table S4. Analysis of amino acid species and contents before and after degradation.

**Amnio acid**	**0 h/(mg/L)**	**48 h/(mg/L)**
Val	5.90±0.34	575.89±11.26
Phe	2.68±0.08	361.37±9.75
Leu	0	257.93±4.46
Ile	0	232.88±5.18
Ser	6.24±0.22	134.43±1.35
Gly	3.51±0.09	114.36±1.14
Asp	2.40±0.04	107.13±1.19
Glu	9.00±0.11	88.01±0.86
Hypro	50.98±0.78	87.57±1.01
Thr	0	78.58±0.68
Cys	0	67.46±0.76
Arg	0	66.53±0.86
Ala	6.74±0.02	61.50±0.47
Met	0	54.07±0.63
His	0	21.30±0.43
Lys	0	20.79±0.35
Tyr	8.79±0.13	0.00
Pro	3.11±0.02	0.00
Total	99.35±3.25	2329.80±26.77

Table S5. Preparation table of main reagents.

**Reagent**	**Compounding methods**
4 mol/L TCA	32.678 g TCA dissolved in 50 mL water
0.5 mol/L Na_2CO_3	5.3 g Na_2CO_3 dissolved in 100 mL water
0.01 mol/L Tris-HCl （pH7.5）	50 mL 0.1 mol/L Tris and 40.6 mL 0.1 mol/L HCl were diluted to 500 mL, and the pH was adjusted to 7.5.
2% casein	0.2 g casein was dissolved in 10 mL mixed solution containing 150 μL 2 mol/L NaOH, 2.85 mL water and 0.01 mol/L Tris-HCl (pH 7.5). The pH was adjusted to 7.5.
Barium chromate solution	1.25 g $BaCrO_4$ dissolved in 100 mL 2.5 mol/L HCl
Calcium-ammonia miscible liquids	0.38 g $CaCl_2 \cdot 2H_2O$ dissolved in 100 mL 6 mol/L ammonia solution and kept away from light.
Sodium mercuric chloride solution	1.36 g Cl_4HgNa_2 and 0.6 g NaCl dissolved in 100 mL water, filtered
1.2% Ammonium sulfamate	1.2 g Ammonium sulfamate is dissolved in 100 mL of water.
0.02% Pararosaniline hydrochloride	0.1 g of Pararosaniline hydrochloride was dissolved in 100 mL of water, 20 mL was taken out, and mixed with HCl 1:1 to make the solution color change from red to yellow, and water was added to constant volume to 100 mL.
Formaldehyde-pararosaniline solution	0.2 % formaldehyde solution and 0.02 % pararosaniline hydrochloride solution were mixed in equal volume.
10 mol/L DTNB	0.396 g DTNB was dissolved in 100 mL pH 7.0 PBS buffer and stored at 4 °C in dark.

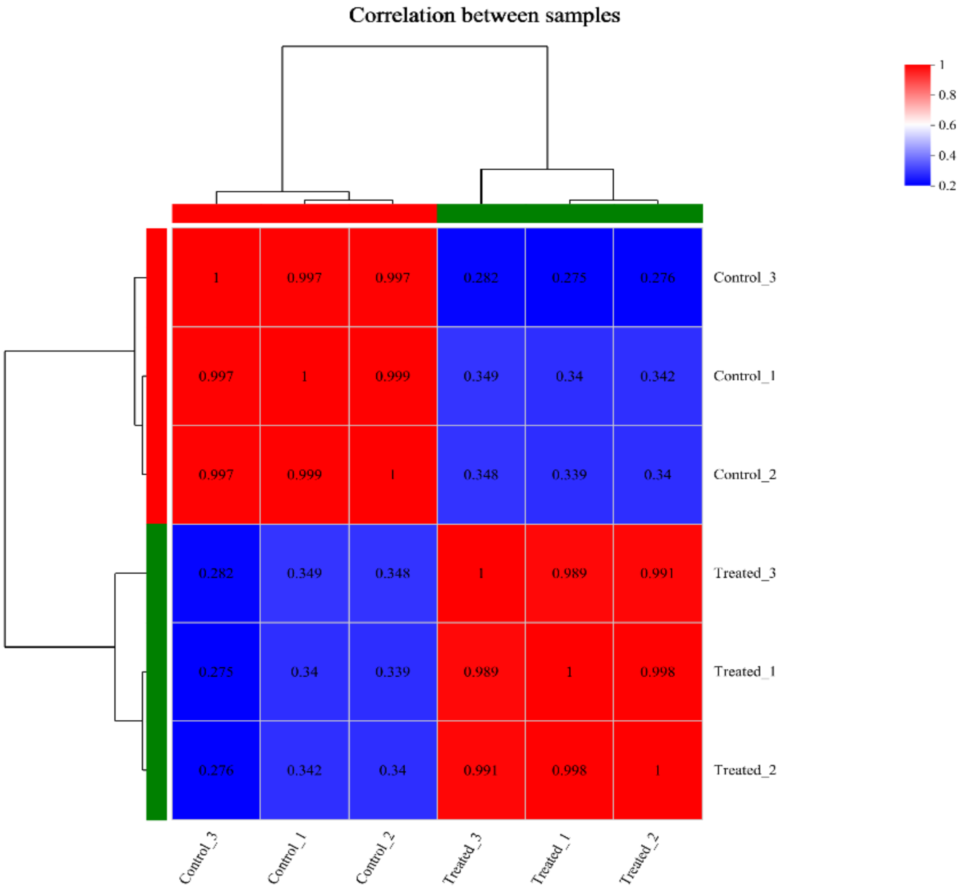

Figure S1. An analysis of the sample correlation heat map. The distance between each sample point indicates the similarity between samples, with closer distances suggesting higher similarity. The horizontal axis represents the contribution of the first principal component (PC1) in distinguishing samples, while the vertical axis represents the contribution of the second principal component (PC2) in distinguishing samples.

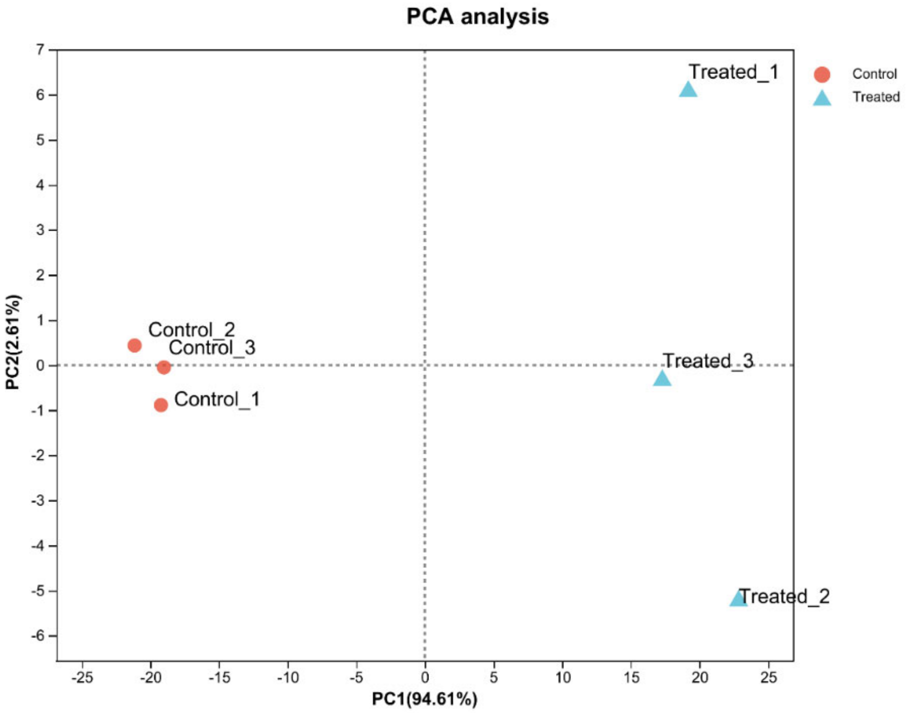

Figure S2. Principal Component Analysis (PCA) correlation analysis was conducted to examine the correlation levels between samples. In the figure, sample names are displayed on both the right and lower sides, while sample clustering is shown on the left and upper sides. The squares in varying colors indicate the correlation levels between the pairs of samples.

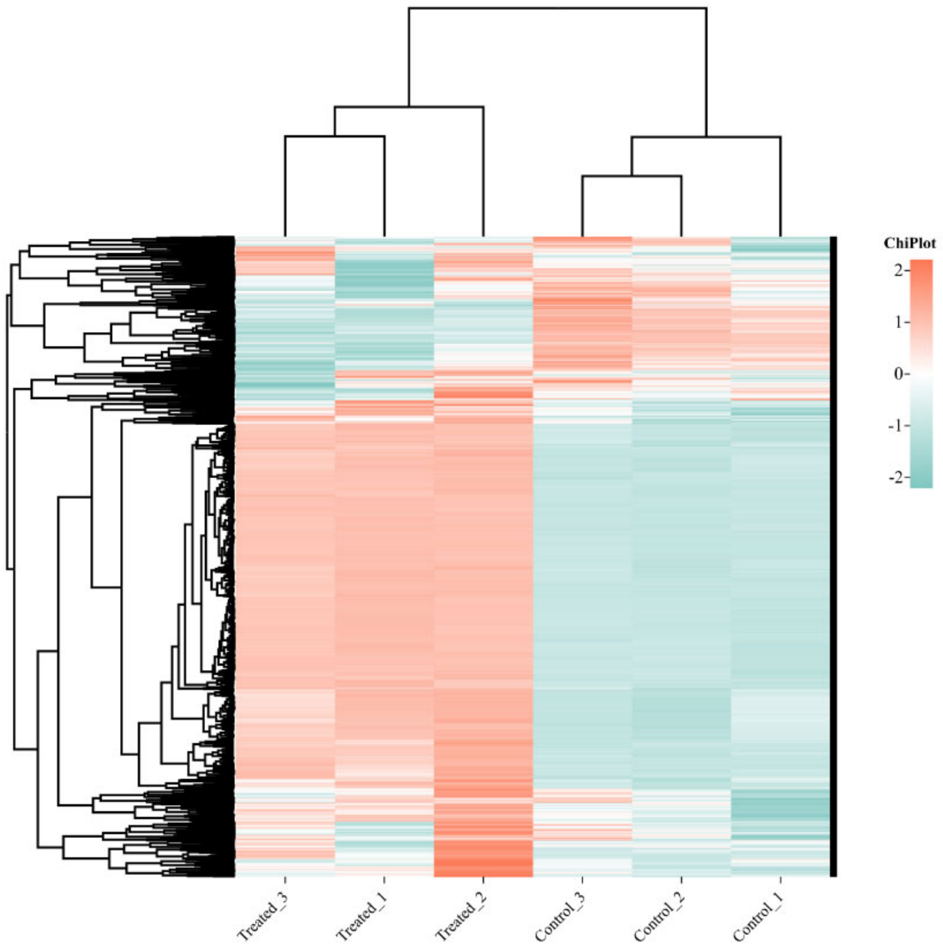

Figure S3. Gene cluster heatmap analysis. Each column represents a sample, and each row represents a gene. The heat map uses color depth to represent the gene's expression level in each sample. On the left side of the diagram, a tree diagram illustrates gene clustering. The closer the branches of two gene clusters are, the more similar their expression levels are. At the top of the diagram, another tree diagram displays sample clustering, with color blocks representing different groups. At the bottom, sample labels are displayed. The closer the branches of two sample clusters are, the more similar the expression patterns of all the genes in the samples are, indicating a closer trend in the changes of gene expression levels.

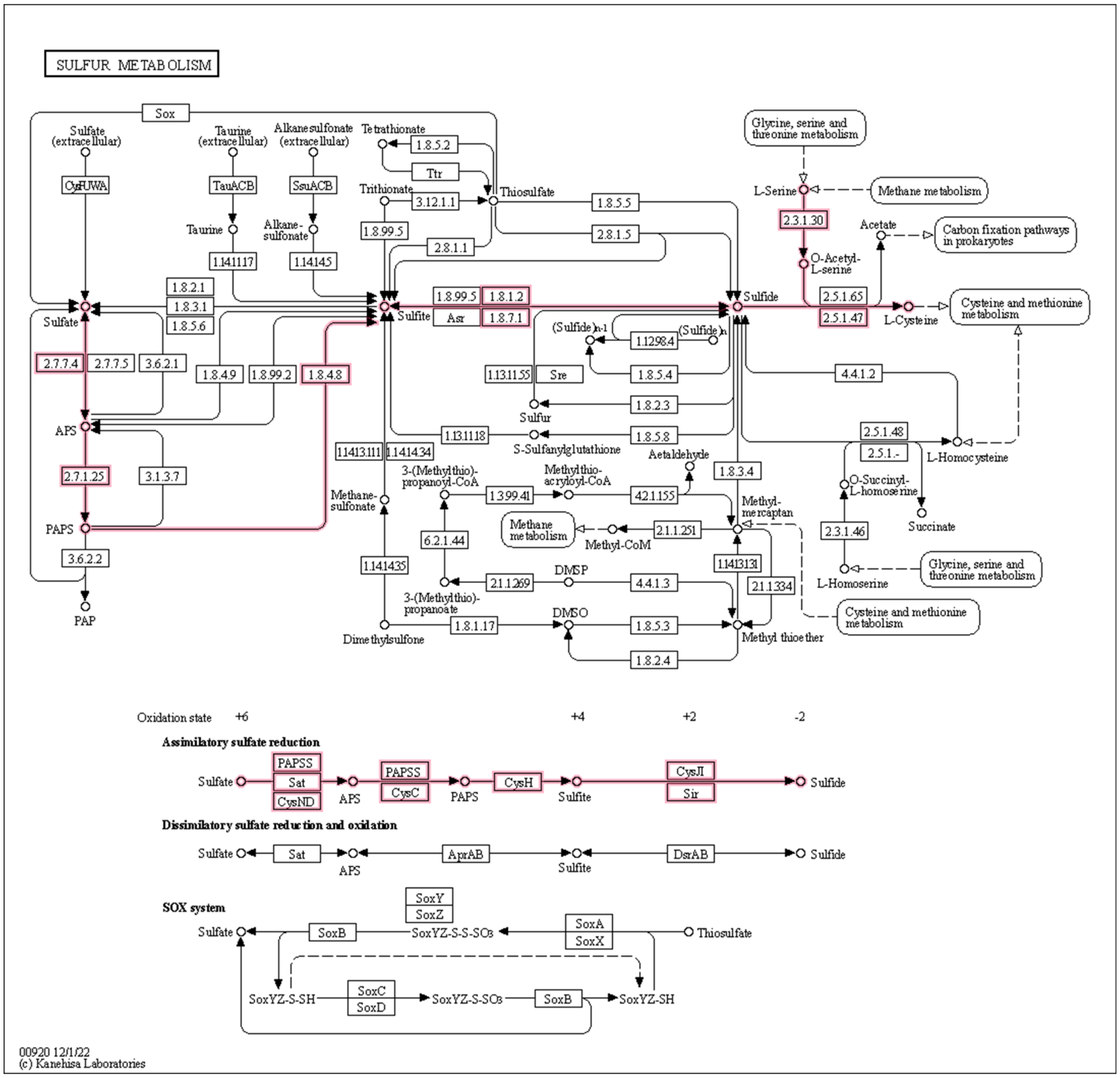

Figure S4. Schematic representation of sulfite metabolism.

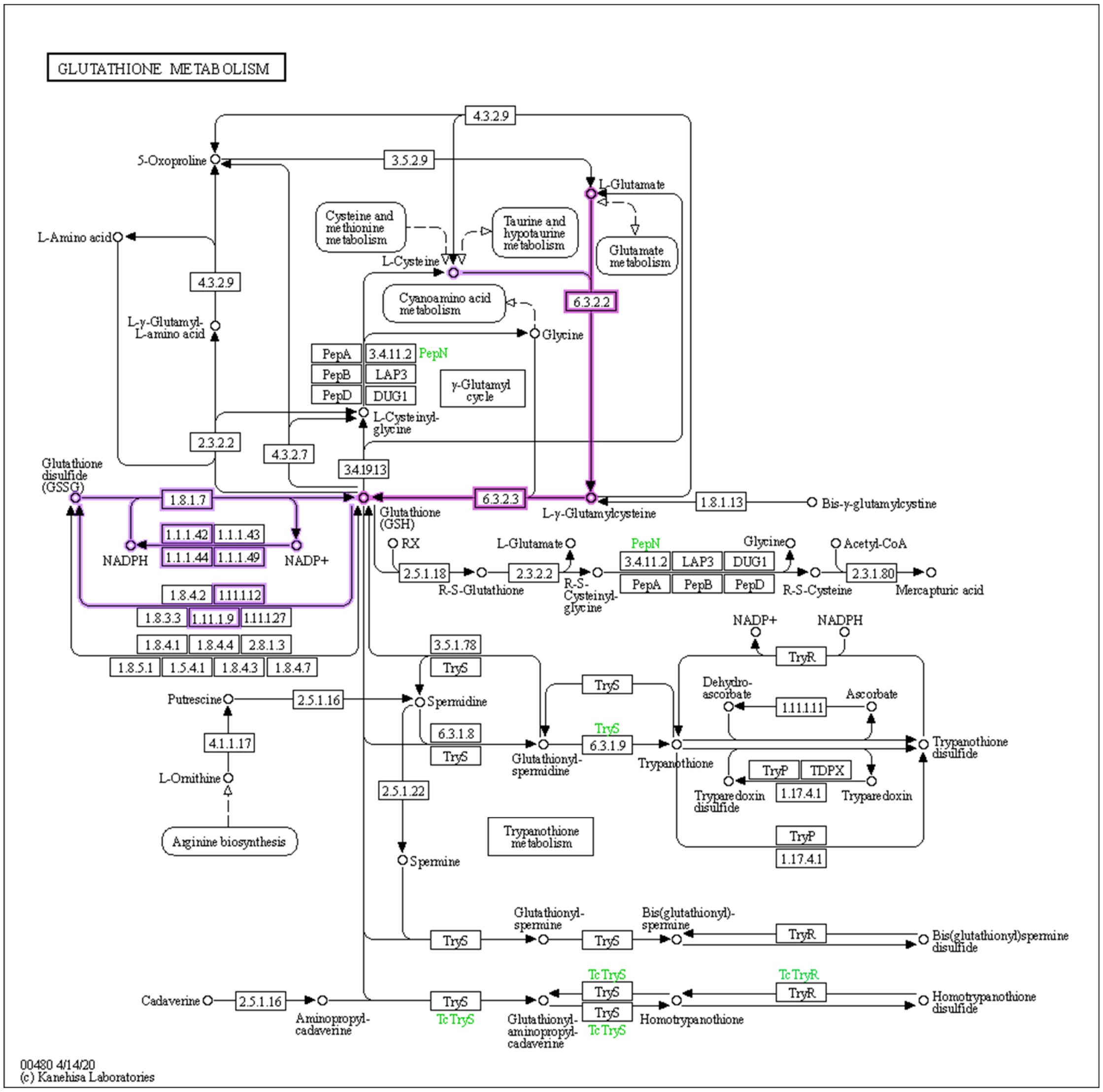

Figure S5. Schematic representation of GSH production.
